# Spatial organization of bacterial populations in response to oxygen and carbon counter-gradients in pore networks

**DOI:** 10.1038/s41467-018-03187-y

**Published:** 2018-02-22

**Authors:** Benedict Borer, Robin Tecon, Dani Or

**Affiliations:** Department of Environmental Systems Science, ETH Zürich, Universitätstrasse 16, 8092 Zürich, Switzerland

## Abstract

Microbial activity in soil is spatially heterogeneous often forming spatial hotspots that contribute disproportionally to biogeochemical processes. Evidence suggests that bacterial spatial organization contributes to the persistence of anoxic hotspots even in unsaturated soils. Such processes are difficult to observe in situ at the microscale, hence mechanisms and time scales relevant for bacterial spatial organization remain largely qualitative. Here we develop an experimental platform based on glass-etched micrometric pore networks that mimics resource gradients postulated in soil aggregates to observe spatial organization of fluorescently tagged aerobic and facultative anaerobic bacteria. Two initially intermixed bacterial species, *Pseudomonas putida* and *Pseudomonas veronii*, segregate into preferential regions promoted by opposing gradients of carbon and oxygen (such persistent coexistence is not possible in well-mixed cultures). The study provides quantitative visualization and modeling of bacterial spatial organization within aggregate-like hotspots, a key step towards developing a mechanistic representation of bacterial community organization in soil pores.

## Abstract (web summary)

Spatial organisation of bacteria could contribute to the persistence of anoxic hotspots in soils, however such processes are difficult to observe directly. Here, the authors develop an experimental platform mimicking resource gradients postulated in soil aggregates to assess bacterial spatial organisation.

## Introduction

Soil is a living and evolving ecosystem, hosting the greatest abundance and diversity of microbial life in the biosphere and serving numerous regulatory and provisional functions essential for life^1,2^. Microbes are at the core of soil ecological functioning; they drive key biogeochemical cycles of carbon and nitrogen, and regulate other fluxes important for plant function^3^. The dynamics, composition, and distribution of soil microbes are shaped by heterogeneous water and resource distribution, and by their ability to rapidly adapt to dynamic changes in local conditions^4^. These intricate local interactions give rise to dense biological hotspots^5^ that often coincide with nutrient-rich zones such as the rhizosphere^6,7^, detritusphere^8^, biopores^9–11^, and soil aggregates^5,12–14^. Microbial activity in hotspots accounts for a large portion of soil biogeochemical fluxes, for example, the production of nitrous oxide within a soil core is dominated by denitrification hotspots in which 1% of the soil volume may account for up to 90% of the denitrification activity^15^. The localized and intense microbial activity within small soil volumes gives rise to the formation of anoxic regions. Diffusing oxygen is consumed directly at the periphery of such hotspots^16,17^, which in turn promotes segregation of aerobes and anaerobes within the local soil microbial community^18^. This spatial organization of bacterial cells combined with high density of microbial activity promotes and sustains anoxic zones even in aerated soils^19^, for example, in drained rice paddy soils^20^ or within the rhizosphere^17^. Soil aggregates have received considerable attention in the past^14^, concerning their internal chemical conditions^16,21^, and the distributions of carbon^22,23^ and bacterial communities^24–26^.

The formation of microbial community spatial patterns at relevant (micro)scale has been studied over the past decades, with several recent mechanistic models^27–30^ and experiments^31–35^, highlighting the importance of spatial organization processes and functional implications^26^. Here, spatial organization represents the emergence of microbial community patterns that stem from local interactions^36^. These recent studies have hinted at key factors that promote spatial organization of microbial consortia, including (but not limited to) nutrient spatial gradients, trophic dependencies, and chemotactic cellular motility^37^. Evidence suggests that microbial communities within soil aggregates spatially organize in response to diffusion in three-dimensional pore networks with biologically mediated oxygen gradients^16,21^. The potential role of persistent and opposing gradients (hereafter counter-gradients) of oxygen and carbon on microbial function has been demonstrated experimentally by glucose perfusion (aimed to disrupt established carbon gradients) with marked changes in microbial activity for different aggregate sizes^24^. These observations are supported by studies of microbial community composition that show organized species distribution at the pore scale^25,38,39^. Despite the importance of such microscale spatial organization processes, evidence of bacterial organization in soil pores remains elusive due to the opaque nature of soil particles and difficulties of in situ visualization. Stable spatial patterns of microbes can emerge from an initially homogeneously distributed community as the result of differential growth within a heterogeneous habitat. Studies documenting microbial community patterns in soil often do not discriminate between spatial organization driven by cell motility or by preferential growth and cell division, owing to the lack of control over initial microbial distributions^25,31,40–43^. Yet, such a distinction is of paramount importance for the temporal dynamics of bacterial hotspots, especially considering the highly dynamic and episodic wetting of soil (and establishment of nutrient gradients).

Notwithstanding the growing number of observations from field studies, the inherent diversity and complexity of soil microbial life in a multifaceted environment remain entangled and limits systematic hypotheses testing of biophysical processes and validation of mathematical models. To overcome these limitations, we propose to investigate a well-characterized microbial community in simple and reproducible artificial systems that nevertheless capture salient features of the soil habitat. Although significantly simplifying, the full scope of soil–microbe complexity, such a bottom-up approach, permits isolation of certain key soil physicochemical components, making them amenable for systematic investigation towards extracting general principles for microbial organization in soil pores.

The hypothesis is that bacterial communities composed of members with different metabolic capabilities (and trophic preferences) that reside within a structured environment (pore networks) would spatially organize over time in response to nutrient counter-gradients. To study basic aspects of bacterial community spatial organization, we have developed an experimental platform that retains essential features that develop in soil aggregates: water-filled micrometric pore networks that supports persistent counter-gradients of carbon and oxygen. The system is comprised of sealed glass-etched pore networks with access at five locations (ports). A mathematical model of the experimental system was developed to interpret and gain insights into the experimental results. The model combines numerical nutrient diffusion with an individual-based representation of bacterial cells and enabled us to simulate scenarios that were impossible to create within the experimental model system such as ubiquitous oxygen. We selected a model bacterial community composed of an obligate aerobe and a facultative anaerobe, both able to disperse by swimming motility and to colonize the pore networks. The study aimed to document counter-gradient induced soil bacterial spatial organization in action, by direct visualization in pore networks and by mechanistic modeling of key processes. We have shown that aerobic and anaerobic bacterial populations spontaneously segregated in response to pore geometry and nutrient gradients. Counter-gradients of oxygen and carbon were necessary and sufficient for the resulting segregation, as demonstrated by experiments and simulations. We demonstrated that while a structured habitat with gradients promoted the stable coexistence of the two bacterial species, their growth in well-mixed habitats led to the competitive exclusion of one of the species.

## Results

### Pore networks to study organization of bacterial populations

**Fig. 1:**
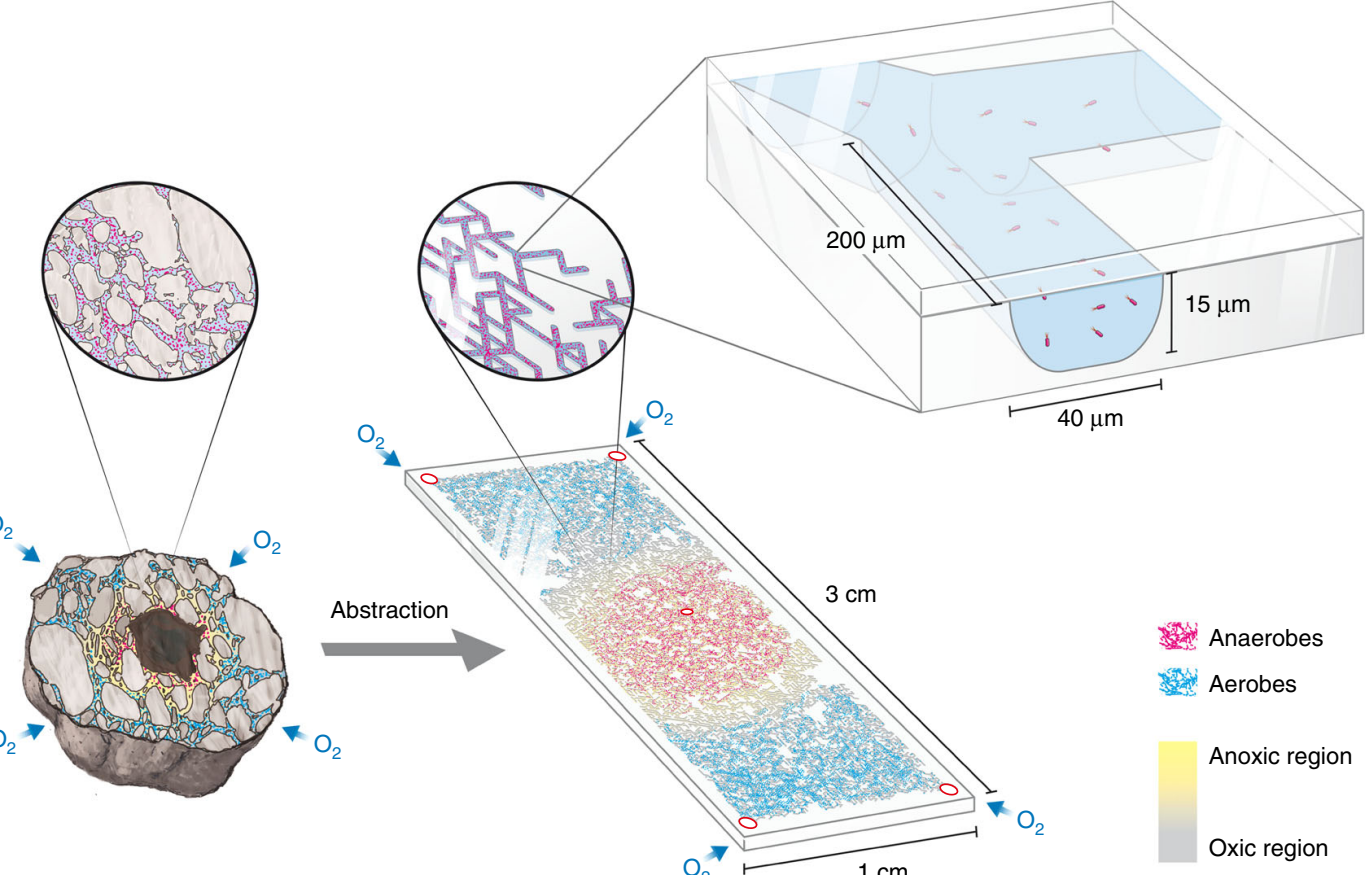
Conceptual view of a model pore network. Aerobes (blue) and anaerobes (red) spatially segregate in a natural soil aggregate due to anoxic conditions at the core (illustrating a central carbon source and oxygen diffusing from the aggregate outer surface). Glass-etched micrometric 2-D pore networks mimicking an idealized cross-section in a soil aggregate were used in the experiments. Chemical boundary conditions similar to those postulated for saturated soil aggregates within an unsaturated soil were imposed via 5 ports allowing access to an otherwise sealed pore network. The pore network geometry allows nutrient diffusion through saturated pores and ample space for bacterial flagellated motility. The emerging spatial self-organization of the two microbial species due to opposing nutrient sources and locally modified gradients can be directly visualized and quantified using fluorescence microscopy through the transparent glass walls

**Fig. 2:**
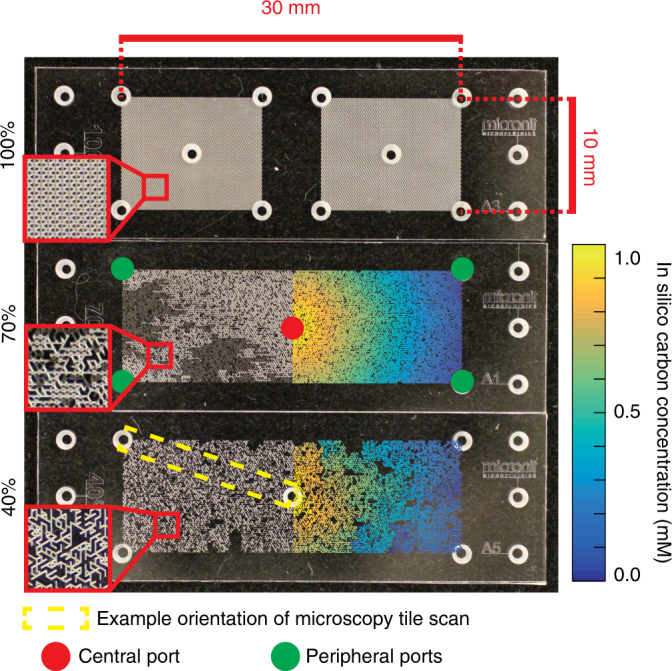
Images of the micrometric pore networks with varying levels of pore connectivity. The pore network connectivity was systematically reduced by randomly deleting channels (30% and 60% of the channels deleted in the 70% and 40% lattices, respectively) while ensuring that the network remains connected (maintains percolation across opposing sides). Four peripheral ports and one central port allow access to the otherwise hermetically sealed network. Overlaid colored networks show in silico simulated carbon gradients emerging 1 day after bacterial growth with carbon source in the central port and peripheral oxygen (with aerobes and anaerobes present in the network). The network geometry exerts a significant influence on network-wide concentration fields as shown with the overlaid carbon concentration. Microscopy images of the glass-etched micromodels with inoculated bacterial cells were taken from the central to a peripheral port (dashed yellow rectangle)

The experimental set-up, consisting of micrometric pore networks etched in glass, was designed to allow control of physicochemical conditions and observation by microscopy (Fig. 1). The network of 200 μm × 40 μm × 15 μm (*L* × *W* × *H*) rectangular channels (bonds) permits flagellated rod-shaped bacteria to freely disperse in the pore network by means of swimming motility. The pore network was saturated with aqueous medium (modified M9 minimal medium, see Methods) that inhibited oxygen gas diffusion while enabling rapid diffusion of solutes (carbon and nitrogen compounds). Five ports allowed access to the otherwise hermetically sealed channel network to impose boundary conditions mimicking resource architectures postulated for carbon-centered hotspots such as soil aggregates (Fig. 2). The model pore network facilitates observation and quantification of bacterial spatial organization as a function of network geometry and resource gradients.

The pore networks architecture was varied to represent different degrees of pore connectivity that would alter diffusional and cell dispersion pathways (resembling aqueous habitat fragmentation in unsaturated soil). A fully connected network (each junction/node is connected to six channels) was progressively disconnected by random deletion of channels while maintaining backbone connectivity (i.e., each channel of the network is accessible from every location within the network) (Fig. 2). We expected that regions in the pore network would become anoxic due to the combined effects of low oxygen diffusion via saturated pores and oxygen consumption by (aerobic) bacteria.

The experiments were guided by a mathematical model that combined nutrient diffusion with an individual-based representation of bacterial cells^30,44–46^. The spatial characteristics of the experimental pore networks were reproduced in the mathematical model for scenario generation. Bacterial cells were represented in the model by motile agents capable of chemotactic motion in response to nutrient and oxygen gradients^30,47^. The individual agents (bacterial cells) interacted with their chemical environment by sensing and consuming local nutrients and were attracted or repelled by oxygen concentrations. The virtual bacteria grew following Monod kinetics; locally adjustable growth rates, and the ability to divide or deplete internal energy and die were represented (see Methods for details of the model)^30,44,48–50^. Nutrient boundary conditions within the mathematical model are congruent to the respective experimental scenario. Additionally, different scenarios such as neglecting chemotaxis or removing nutrient gradients were simulated to identify key factors governing spatial organization of the model community in silico.

For the experiments, we have used two motile bacterial species isolated from soil, an obligate aerobe *Pseudomonas putida* and a facultative anaerobe *Pseudomonas veronii*, as the model community. The two species were tagged with autofluorescent proteins (using a green and a red fluorescent protein for *P. putida* and *P. veronii*, respectively) to permit their identification and distribution in the pore network. The formation of stable spatial bacterial community patterns, in response to the arrangement of carbon and oxygen resources, were quantified from digital image analyses of fluorescence microscopy images. The two species were simulated in the mathematical model as an obligate aerobe and an obligate anaerobe. *P. veronii* was represented as obligate anaerobe due to limitations of modeling facultative anaerobes using simple growth kinetics. For simplicity, *P. putida* and *P. veronii* are further referred to as (obligate) aerobes and (facultative) anaerobes, respectively.

### Counter-gradients drive segregation of bacterial populations

**Fig. 3:**
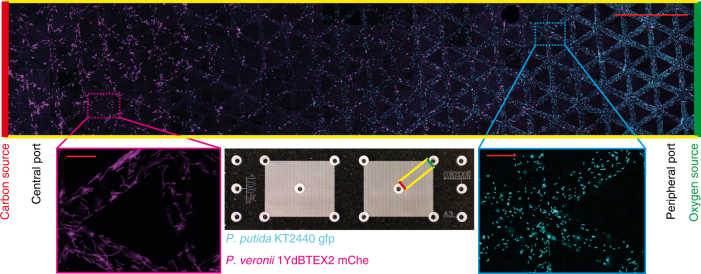
Experimentally observed spatial segregation of bacterial populations in a micrometric pore network. The facultative anaerobic *Pseudomonas veronii* strain 1YdBTEX2-mChe is tagged with the mCherry fluorescent protein and shown with pseudo-color magenta, and the obligate aerobic *Pseudomonas putida* strain KT2440-gfp is tagged with the eGFP fluorescent protein and shown with pseudo-color cyan. The two species were mixed 1:1 in suspension and inoculated to the central port (total of ≈500 cells per species) of a pore network with a carbon source (citrate) located at the center and oxygen diffusing from the peripheral ports. Both species were able to disperse through the pore network by flagellated motility. The exemplary overlay fluorescence image (top) shows the distribution of the two bacterial populations in a transect across the 100% connected pore network (see inset) 1 week after inoculation (image contrast and brightness were enhanced for better visualization of the bacterial populations). The scale bar represents 500 μm. The two close-up images show individual bacterial cells within one microscopy image, as used for digital image analysis and cell counting (each scale bar represents 40 μm)

**Fig. 4:**
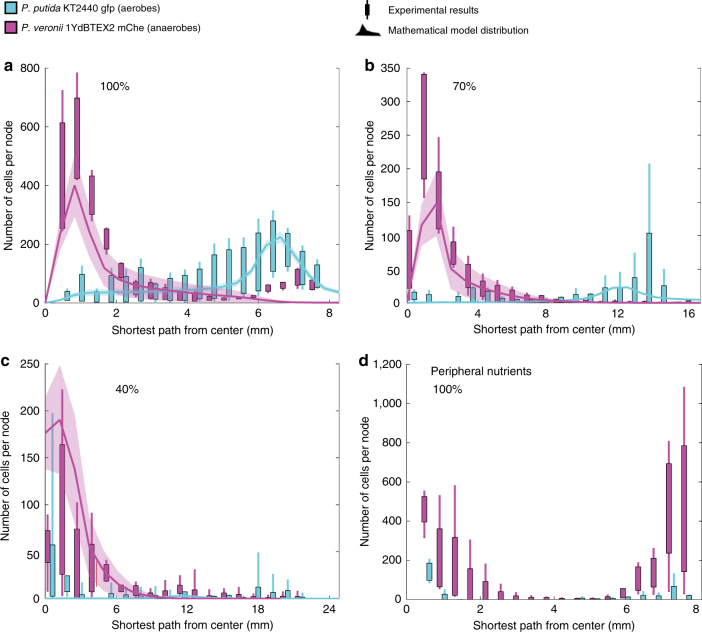
Distribution of aerobes and anaerobes as a function of spatial positioning within the different pore networks. Results show combined experimental cell distribution and simulation results from the mathematical model for all pore network connectivities (**a**–**c**), using counter-gradients of citrate (maximum concentration at center port) and oxygen (maximum concentration at peripheral ports). Experimental results are represented in boxplots (whiskers indicate minimum to maximum value of data) of three, four, and five experimental replicates for the 100%, 70%, and 40% pore networks, respectively. In the mathematical model results, thick line indicates the mean and shaded area includes 95% of all cells. In the experiments, bacterial cells in fluorescence microscopy images were identified using digital image analysis, mapped onto the pore network, and attributed to the closest nodes. These were subsequently grouped into bins with respect to their shortest path from the central port. A pore-scale segregation of the two species is visible within each connectivity. In the 40% lattice, the distribution is less clear due to perturbations of the pore network geometry. To test the role of counter-gradients, the experimental results in **d** depict bacterial distributions for carbon and oxygen collocated at the peripheral ports (eliminating counter-gradients) resulting in dominance of facultative anaerobes at both the central and peripheral ports. Simulations of this scenario would result in the dominance of aerobes at the peripheral port due to the (simplified) representation of *P. veronii* as an obligate anaerobe

Prior to bacterial inoculation, the pore networks were saturated with modified M9 minimal devoid of citrate overnight under vacuum. An intermixed community containing a 1:1 ratio of aerobes to facultative anaerobes was inoculated by pipetting at the central port (Fig. 2), which was subsequently sealed using an agar plug containing a high concentration of citrate (carbon and energy source) and nitrate (electron acceptor for anaerobic respiration), and finally capped with silicone to prevent oxygen diffusing into the port. After seven days of incubation at room temperature (~23 °C), the distribution of the two species within the pore networks was imaged using a fluorescence microscope (Fig. 3). Image analysis tools were used to locate bacterial cells within the microscopy tile scans and consequently mapped onto the pore network for direct comparison with model predictions. This step of mapping bacterial cells onto the network also enables their distribution to be related to network properties such as the shortest path distance (Supplementary Fig. 1), which is important since diffusion within the network follows the shortest paths (irrespective of Euclidean distances). The spatial distribution of the two bacterial species along the shortest path from the central to peripheral ports, including predictions from the mathematical model, is shown in Fig. 4.

While chemical gradients within the network were not observable in these studies, we verified the onset of oxygen gradients within the network using oxygen optodes^51,52^. Results confirmed the presence of anoxic conditions at the central port, and of oxic conditions at peripheral ports (in contact with atmospheric oxygen) (Supplementary Fig. 2). A control experiment with a network containing no bacterial cells confirmed persistent oxic conditions at all five ports (Supplementary Fig. 2), indicating that the presence of anoxic conditions at the center of the pore network was induced by bacterial respiration.

For all three lattice connectivity designs considered here (Fig. 2), the facultative anaerobes dominated the central region of the pore network (close to the carbon source) at the end of the incubation period (Figs. 3 and 4). Clearly distinguished patterns of spatial segregation of the two bacterial populations were visible within the 100% and 70% connectivity lattices with obligate aerobes residing closer to the peripheral ports (Figs. 3 and 4a, b). The segregation pattern was somewhat obscured within the 40% lattice (Fig. 4c). The mathematical model captured salient features of the observed spatial segregation (between aerobes and anaerobes) for the different connectivity pore networks, although the position of maximal aerobe proliferation was predicted closer to the peripheral ports than observed (Fig. 4). In the pore networks of highest connectivity (100% lattice, Fig. 2), a subpopulation of facultative anaerobes was experimentally observed close to the peripheral port with a mean magnitude comparable to that of the obligate aerobes, while the mathematical model predicted only aerobes at this position (Fig. 4a). The size of the aerobic population close to the peripheral port was much smaller in the pore network with intermediate connectivity (70% lattice, Fig. 2) compared to the 100% lattice, and no secondary population of facultative anaerobes was observed. In line with the experimental results, the mathematical model predicted a comparable segregation of aerobes and anaerobes (Fig. 4b) with obligate aerobes closer to the peripheral port. The spatial pattern within the lowest connectivity lattice was less obvious due to network structural heterogeneity and localization of aerobic cells within the network (Fig. 4c). More specifically, each imaging transect covered multiple regions of the network, some of which were more or less connected to each other, resulting in patchy distributions. The mathematical model captured the ratio of anaerobic to aerobic population size well, but predicted a more defined peak of anaerobic cells than was shown in the experimental results (Fig. 4c). A significant reduction in total bacterial cell density (two-sample *t* test, two-tailed) is observed with decreasing network connectivity (*p* values of 0.044 and 0.028 when comparing the fully connected network to the 70% connectivity lattice and the latter with the 40% connectivity lattice, respectively). The same comparisons based on the mathematical model results are highly significant with *p* values of 9.82 × 10^−10^ and 1.75 × 10^−5^. Biomass differences within the mathematical model are more significant due to the higher number of numerical realizations compared to the number of experimental replicates. We conducted a verification experiment to confirm that the observed spatial organization was due to the opposing carbon and oxygen gradients using a fully connected lattice with collocated oxygen and carbon sources at the peripheral ports while bacterial cells were introduced via the central port. The facultative anaerobe *P. veronii* dominated both the central and peripheral ports in the collocated scenario (Fig. 4d). Despite the higher growth rate of obligate aerobic species in fully oxic environments, the initial conditions within the network (anaerobic-limited or oxygen diffusion-limited conditions) favored the facultative anaerobes, giving them a competitive edge during the early stage of community establishment, thus enabling them to outcompete the obligate aerobic species even at the periphery.

In addition to the experimental results, model simulations have been used to study how previously identified factors (chemotactic behavior, cell motility, resource gradients, and metabolic preferences^28^) govern bacterial spatial organization at the pore scale. The main aim of these simulations was to obtain bacterial distributions for scenarios that could not be easily tested in the experimental pore networks. These included the omission of cell chemotactic behavior and prevention of substrate gradients within the network. Both of these scenarios resulted in a uniform distribution of bacterial cells along the shortest path from the center to the periphery, as shown in Supplementary Fig. 3.

### Segregation favors coexistence of competing species

**Fig. 5:**
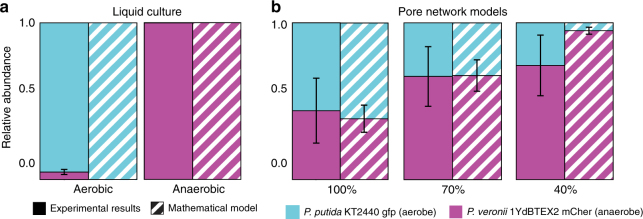
Relative abundance of the two species in the community as a function of growth conditions and spatial structure of the environment. **a** Aerobes and facultative anaerobes were cocultured in shaken liquid medium to create an unstructured environment. A 1:1 ratio of aerobes to anaerobes was used to inoculate sealed serum flasks (to promote anaerobic respiration) or test tubes (aerobic respiration). After 72 h of incubation, culture samples were plated on selective agar media to determine the ratio of aerobes and anaerobes. Error bars show one standard deviation around the mean calculated from ten replicate cultures. Simulations represented a fully oxic and fully anoxic environment for the aerobic and anaerobic conditions, respectively. **b** Relative abundance of aerobes and anaerobes in pore networks calculated from results presented in Fig. 4a–c using counter-gradients (central carbon and peripheral oxygen). Error bars represent one standard deviation around the mean calculated from eight model realizations for mathematical results and three, four and five replicates for the experimental results in the 100%, 70% and 40% connectivity, respectively. In well-mixed environments represented as liquid flasks, very limited coexistence (aerobic conditions) or absence of coexistence (anaerobic conditions) were observed with dominance of obligate aerobes in the aerobic case and vice versa for the anaerobic case (**a**). Coexistence was facilitated due to the structured environment and resulting pore-scale segregation of the two species (**b**)

Our experiments demonstrated that competing bacterial populations of aerobes and (facultative) anaerobes attained a state of stable coexistence, suggesting an important role of spatial segregation within pore networks (that limit nutrient diffusion pathways) in promoting such coexistence. We examined the question of whether aerobes and facultative anaerobes would also coexist in the absence of a spatially structured environment (pores or channels) and in the absence of nutrient gradients. We inoculated the two species in sealed serum flasks containing liquid medium for anaerobic growth conditions. In both, experimental and simulation results, the obligate aerobes clearly dominated the community under aerated growth conditions (95% of total population), while conversely the facultative anaerobes prevailed under anoxic conditions (99.9%) (Fig. 5a). These results contrasted strikingly with the coexistence observed in micrometric pore networks (Fig. 5b), where a reduction in connectivity increases the relative abundance of facultative anaerobes within the community (39 ± 21%, 66 ± 19%, and 95± 19% of the community were anaerobes for the 100%, 70%, and 40% lattices, respectively), which was mainly due to a decrease in obligate aerobes. The mathematical model captured these characteristics reasonably well, although it predicted lower relative abundance of obligate aerobes compared to the experimental results (Fig. 5b). Furthermore, additional pore network geometries were created to systematically evaluate interactions between network connectivity and relative abundance of the two species using the individual-based model (see Supplementary Fig. 4). The main effect of network connectivity stems from a reduction in network diffusivity that translates to lower biomass for the aerobic species (as they rely on carbon diffusing from the core) while the obligate anaerobe benefits from collocated sources, resulting in a gradual shift in relative abundance.

## Discussion

This study reports visualization and quantification of spatial organization of a two-member bacterial community in pore networks mimicking the architecture and nutrient gradients postulated in carbon-centered hotspots such as soil aggregates (i.e., a carbon-rich anoxic core and oxic exterior boundaries) (Figs. 1 and 2). The experiments and model simulations were used to test the hypothesis that nutrient counter-gradients and cell motility within pores were necessary and sufficient conditions for the spatial organization and stable coexistence of bacterial species with different respiratory metabolisms.

The two species were able to disperse and grow in the pore networks, coexisting as largely spatially segregated populations with the facultative anaerobes dominating the carbon-rich and anoxic central region and aerobes prevailing in the well-aerated peripheral regions (Figs. 3–5). In agreement with observations (Supplementary Fig. 2), the mathematical model predicted that bacterial aerobic respiration would rapidly deplete oxygen concentrations in the pore network, resulting in an oxygen gradient from the oxic exterior to the mostly anoxic central region dominated by anaerobes (Fig. 4 and Supplementary Fig. 5). However, the oxygen gradient was not sufficient to maintain the segregation of aerobes and anaerobes: the presence of a carbon (citrate) counter-gradient (i.e., high in the center and low at the periphery) was also required (Fig. 4). Additional simulations were performed to confirm the necessity of gradients for spatial organization and coexistence of the two species. Complete removal of gradients within the networks was infeasible experimentally since bacterial communities self-engineer their environment through consumption of nutrients. Removing nutrient gradients in the mathematical model (by homogenizing the network with nutrients at each time step) resulted in a uniform distribution of aerobes and anaerobes (Supplementary Fig. 3). These results underline the importance of oxygen-carbon counter-gradients for spatial organization of aerobic and anaerobic populations, which was previously posited for microbial organization in soil aggregates^16,53^. The glass pore networks mimicking soil aggregate cross-sections were used in this study as a proxy for microbial hotspots where counter-gradients emerge due to spatially isolated nutrient sources. This architecture is not unique to soil aggregates, but is commonly found in soil; for instance, within the rhizosphere (rhizodeposits as carbon source)^54^ or detritusphere (dead root matter/fungi)^5^. Such counter-gradients have also been documented within the diffusive regime of marine snow particles during nighttime, when oxygen production by photosynthesis ceases^55^.

To test how pore network topology and the resulting diffusional landscape affect bacterial organization, we varied the aqueous connectivity in the network from a homogeneous lattice (100%) to a heterogeneous and poorly connected lattice (40%) (Fig. 2). Total bacterial abundance decreased with a reduction of pore network connectivity (Figs. 3 and 4), suggesting sensitivity to diminishing carbon fluxes due to fragmented and tortuous diffusive pathways. Similarly, the mathematical model predicted lower total carbon fluxes from the central port to the periphery with decreasing pore network connectivity (Fig. 2 and Supplementary Fig. 5). Decreasing pore network connectivity modifies and obscures the pattern of aerobe and anaerobe segregation (Fig. 4). Low connectivity (40%) resulted in a patchy distribution of aerobes along the shortest path from the center in both experiments and model simulations. We could not ascertain whether the aerobes observed near the central region in the low connectivity network of the experiment were active or dormant (pores are inaccessible for direct sampling). However, since *P. putida* is an obligate aerobe and the pore network’s core was mostly anoxic as mentioned above, we surmise that the remaining aerobic cells at the central port were metabolically inactive. Overall, the results showed how the change in diffusional landscape dictated by the connecting aqueous phase affects bacterial spatial organization.

In the absence of bacterial activity, molecular diffusion would mix and dissipate nutrient counter-gradients over time (days in our system) (Supplementary Fig. 2). Moreover, the formation and maintenance of oxygen gradients by bacterial respiration created new niches that could be exploited by the facultative anaerobes in the pore network (Fig. 4), whereas under fully oxic conditions the anaerobic population waned (Fig. 5). The observation in the model micrometric pore network thus represents an interesting case of bacterial niche engineering^56^ similar to a mechanism postulated to sustain anoxic hotspots in soils^16^. Additionally, the microhabitats facilitated by the pore network conferred coexistence of the two bacterial species (Figs. 3 and 4) compared to well-mixed flasks (where gradients are mostly absent) that lead to species competitive exclusion (Fig. 5). This demonstrated that the results of interspecific interactions were modified in the micrometric pore network in comparison to flasks. These observations illustrate the crucial role of structured habitats for the stability of microbial communities^57^.

In the experiments, anaerobes were able to colonize regions near the aerated ports in the fully connected (100%) network (Fig. 4), where diffusional carbon fluxes from the center to the periphery were substantial (large number of diffusive pathways and shorter diffusion lengths). The pattern was different than predicted by the mathematical model which assumed that the virtual obligate anaerobes would be inhibited by oxygen. The model could be expanded to consider facultative anaerobes using different growth kinetic expressions such as switching metabolism (between aerobic and anaerobic^49^). Such adaptation would influence the distribution of the facultative anaerobes and relative abundance of the two species while the underlying nutrient landscape would remain as dictated by the network connectivity (see Supplementary Fig. 6). We opted for the more parsimonious model where facultative anaerobes are represented as obligate anaerobes for simplicity as it captures the primary mechanisms that give rise to spatial segregation and coexistence of the two species reasonably well.

An important ingredient for the spatial organization of the community (in addition to counter-gradients, trophic preferences, and connectivity) is bacterial chemotactic motility. Since the mixed inoculum was introduced at the center of the network, aerobes had to traverse the network towards the peripheral ports to establish subpopulations in the oxic regions. Previous studies have demonstrated the motile behavior^58^ and aerotaxis^59^ of aerobic *P. putida* as well as motility of *P. veronii*^60^. Chemotaxis is important for the rate of spatial organization, where bacterial cells may traverse distances orders of magnitude faster than in the absence of chemotactic-guided motility^50^. Importantly, the mathematical model predicted no spatial segregation in the absence of chemotaxis and aerotaxis during periods comparable to the incubation of the experiments (Supplementary Fig. 3). These findings are consistent with theoretical studies that have identified trophic preferences, persistent nutrient gradients, and motility as factors promoting spatial organization of bacterial communities^28^. Trophic preferences are reflected by the segregation of the two species into two subpopulations where facultative anaerobes obtain a metabolic advantage in the anoxic core^18^. Evidence suggests that the observed organization required active migration by flagellated motion and subsequent population growth. Alternate modes such as directed cell division and passive cell diffusion would be too slow to produce the observed spatial pattern within the time frame of the experiments. This suggests that gradient-guided cell motility was an important ingredient in achieving the spatial distribution observed in our experiments.

For simplicity, we have varied aqueous phase connectivity by removing bonds (Fig. 2). In soil environments, changes in aqueous phase connectivity would be influenced by the onset of unsaturated conditions during wetting and drying cycles^4^ (fully saturated pore networks such as 100% lattice would be short lived). Such transients do not preclude bacterial spatial organization, as these are relatively rapid processes (hours) as the study has shown, a time frame comparable to the duration of very wet soil conditions following rainfall or irrigation. Furthermore, microorganisms can modify the local hydraulic properties of unsaturated soil, by colonizing pore surfaces^61^ or via the production of extracellular polymeric substances^62^. Such modifications may influence drainage and drying characteristics of pore spaces^63^ and prolong favorable conditions for bacterial spatial organization in such hot spots.

In conclusion, the experimental and mathematical models developed in this study successfully identified basic biophysical factors that exert control over bacterial spatial organization and that can be related to natural features found in soil environments. The models allowed us to systematically study some of the prime factors that give rise and facilitate bacterial community spatial organization at the pore scale, hence confirming findings of previous theoretical^28,64^ and experimental studies^16,18,21^. Admittedly, the experimental and modeling approaches used in this study involve simplification of the immense biological, chemical, and physical complexities found in soil that operate over a range of time scales to bring about bacterial community response and organization. Nevertheless, consideration of some of the simplest attributes of soil pore spaces offer useful building blocks for improved understanding of ingredients important for bacterial community functioning in soil. Clearly, further efforts are required to address the temporal dynamics of soil bacterial organization and activity in response to changes in chemical boundary conditions induced by desaturation or similar processes. Platforms combining experimental and individual-based models, with controlled boundary conditions while enabling visualization of the response of tagged bacterial populations, offer a promising tool to further reveal the mechanics and dynamics of bacterial organization and function in microhabitats.

## Methods

### Bacterial strains and culture conditions

*Pseudomonas putida* KT2440 (strict aerobe)^65^ and *Pseudomonas veronii* 1YdBTEX2 (facultative anaerobe)^66^ are chromosomally tagged with a mini-Tn7 transposon^67^ conferring resistance to gentamycin and expressing the enhanced green fluorescent protein (eGFP) or the mCherry fluorescent protein, respectively, under control of a constitutively active P*lac*-derivative promoter. Strain KT2440-gfp was a gift from Arnaud Dechesne (Department of Environmental Engineering, Technical University of Denmark) and is described in more detail in ref. ^67^. Strain 1YdBTEX2-mChe was a gift from Jan Roelof van der Meer (Department of Fundamental Microbiology, University of Lausanne); the mini-Tn7-mCherry genetic construct used to tag strain 1YdBTEX2 is described in more detail in ref. ^68^. Both tagged strains were routinely grown in shaken Luria-Bertani (LB) liquid culture or on LB agar plates at 30 °C with 10 μg/mL gentamycin.

As a minimal medium, we used modified M9 (90.2 mM Na_2_HPO_4_, 8.6 mM NaCl, 18.7 mM NH_4_Cl, 14.7 mM KH_2_PO_4_, 15.1 mM l-asparagine, 2 mM MgSO_4_, 0.1 mM CaCl_2_, 0.1 mM FeCl_3_·6H_2_O, 10 μg/mL gentamycin). Additionally, liquid media were supplemented with trace metals (≈1 μM of CuCl_2_, ZnSO_4_, MnCl_2_, ZnSO_4_, CoCl_2_, NiSO_4_, and Na_2_MoO_4_). When required, for example, for overnight cultures or experiments in shaken flasks, media were supplemented with 10 mM Na_3_Citrate and 10 mM NaNO_3_.

### Pore networks design

Glass-etched micrometric pore networks were manufactured by Micronit Microtechnologies (Enschede, The Netherlands). Pore networks were designed in Mathematica by creating a fully connected, hexagonal lattice with the desired channel length (200 μm) and subsequently randomly deleting the required percentage of all bonds (30% of all bonds for the 70% lattice and 60% of all bonds for the 40% lattice) to obtain the reduced connectivity lattice. Compared to other pore network designs which use geometries based on CT images^63^, our choice of simple geometry enables us to relate observed spatial patterns directly to the diffusional properties of the networks as these can be calculated with ease. Finally, percolation of the network was tested (i.e., ensuring a bacterial cell can move to any point within the network from any location). CAD files were then sent to Micronit Microtechnologies for the creation of the desired micrometric networks through isotropic wet etching. Each channel has dimensions of 200 μm × 40 μm × 15 μm (*L* × *W* × *H*), which allows ample space for bacterial flagellated motility^47,69^.

### Bacterial spatial organization in pore networks

Glass-etched micrometric pore networks were saturated with modified M9 media devoid of citrate using a vacuum desiccator (12 h at 0.1 bar). The choice of saturation method resulted in initially microaerobic conditions within the pore network due to the applied vacuum (Supplementary Fig. 2). Overnight cultures of strains KT2440-gfp and 1YdBTEX2-mChe in minimal medium supplemented with citrate and nitrate were diluted in the same medium to obtain an optical density at 600 nm (OD_600_) of 0.01, after which they were mixed at a 1:1 ratio to serve as an inoculation community. One microliter of the inoculation community (approximately 1,000 cells in total) was subsequently pipetted into the central port filling the port itself. After 30 min of allowing bacterial cells to enter the network, 5 μL molten 1.4% M9 agar (approximately double the total pore volume) containing 100 mM citrate and nitrate was pipetted into the central port as a primary carbon and energy source. After a further 60 min, flowable silicone (Elastosil E41) was used to cover the solidified agar and thereby restrict oxygen diffusing into the pore network center. At the four peripheral ports filled with liquid growth media, oxygen was able to diffuse into the pore network following Henry’s law with atmospheric oxygen above the ports. The inoculated lattice was incubated inside a 50 ml Falcon tube (representing a wet chamber to suppress evaporation) for 7 days at room temperature (~23 °C) to allow for bacterial growth and spatial organization.

### Image acquisition and digital image analysis

Prior to image acquisition, the micrometric pore networks were taken out of the incubation chamber, then the agar plug on the central port was removed, and the port was left open for 30 min. This ensured that oxygen was available in the center of the pore network (oxygen is necessary for the maturation of autofluorescent proteins and hence good fluorescent signal). By observing the cell distribution during this oxygenation period, it was ensured that no reorganization of the population was occurring due to the enhanced oxygen availability at the central port. Images of fluorescent bacteria in the pore networks were obtained with a DM6000B epifluorescence microscope (Leica Microsystems, Heerbrugg, Switzerland) using a 40×/0.60 HC PL Fluotar objective (Leica Microsystems). We sequentially recorded phase contrast images and fluorescence images with a DFC350 FX camera (Leica Microsystems) using an L5 filter cube for eGFP (exciter: 480/40; emitter: 527/30: beamsplitter: 505) and a Y3 filter cube for mCherry (exciter: 545/25; emitter: 605/70: beamsplitter: 565) (Leica Microsystems). Exposure time, gain value, and illumination intensity were optimized independently for eGFP and mCherry signals, with a Gamma function set to 1. The Leica Application Suite (LAS) acquisition software (Leica Microsystems) was used to acquire multiple fields of view and to assemble them into a composite image with the tile scan function of the LAS software. Overlay images of eGFP and mCherry fluorescence were shown with the pseudo-colors cyan and magenta, respectively.

Digital image analysis was performed using the KNIME Analytics platform version 3.2.1 (KNIME.COM AG, Zürich, Switzerland) including the KNIME Image Processing toolbox (KNIP) version 1.5.2. eGFP and mCherry fluorescent images were separately exported from the Leica Application Suite software as tiff files and subsequently loaded into KNIME. The images were binarized using the Triangle threshold method^70^. A morphological operation (open, four connected) was performed to erase tiny bright spots too small to be counted as bacterial cells. A connected-component analysis (four connected) yielded individual areas identified as bacterial cell mass. Individual areas with a size smaller than 5 pixels or larger than 1,000 (approximately 0.1% of the image size) were neglected. Each identified bacterial cell was divided by the median cell pixel size to account for areas representing more than one cell (i.e., overlapping or touching cells). Ratios <1 were counted as one bacterial cell while for ratios larger than one the decimal value was taken. In order to calculate the shortest path distance from the center to the cell location, identified bacteria were mapped onto the network using Matlab version R2016b (Mathworks, Natick, MA, USA). Phase contrast tile scans were used to recover the homography using 12 pass points. Bacterial cells were then attributed to the closest node of the pore network. Cells mapped within a radius of 0.5 mm of a port were neglected, as port wall reflections created artifacts within close proximity of the port. Finally, a histogram of bacterial cells along the shortest path from the center to the node location was created using 20 histogram bins to obtain an average cell number per node.

### Confirmation of anoxia and oxygen gradients

An oxygen optode (PreSens VisiSens A1 Detector unit and AnalytiCal 1 Software, PreSens Precision Sensing GmbH, Regensburg, Germany) was used to assess oxygen levels at the center of the pore network and peripheral ports. This was done by including an SF-RPSu4 oxygen sensor foil on top of the central port before adding nutrient-rich agar and flowable silicone to measure oxygen levels at the center. At the peripheral ports, the same oxygen sensor foil was brought into contact with growth media inside the port (atmospheric oxygen). Oxygen levels were observed after 7 days of incubation at room temperature (~23 °C). The importance of bacterial activity for maintaining the oxygen gradients was assessed by comparing two lattices, where one was inoculated with the standard model community (1:1 ratio of aerobes to anaerobes) while the other was left sterile.

### Experiments in unstructured and homogeneous environment

Overnight cultures of both aerobes and anaerobes in 5 mL modified M9 minimal media were diluted to an OD_600_ of 1. The two species were mixed at a 1:1 ratio as an inoculation community having an equal abundance. Ten microliters of this community were subsequently inoculated into 10 mL of modified M9 medium containing 10 mM citrate and 10 mM nitrate in a test tube (for aerobic growth) or sealed serum flasks (for anaerobic growth). Ten replicate cultures were used for both the aerobic and anaerobic case. Bacterial cocultures were then incubated with shaking at 280 rpm and 30 °C for 48 h. Samples were diluted down to an OD_600_ of 0.1 (approximately 5 × 10^7^ cells per mL). The cultures were then further diluted by a factor of 10^4^ before plating 20 μL onto LB agar containing 10 μg/mL gentamycin. Three plates from each flask/test tube were created. Agar plates were incubated for 48 h at 30 °C and subsequently stored at 4 °C for a further 24 h to allow the accumulation of fluorescent proteins before counting the number of colony-forming units per species.

### Mathematical model description

The mathematical model consists of a combination of numerical nutrient diffusion with an individual representation of bacterial cells. Chemical diffusion is simulated as a one-dimensional diffusive process following Fick’s law between two nodes/junctions while respecting mass balance at a node as described by Eq. 1, where *C* is the substrate concentration, *t* is time, *J* are the fluxes between nodes, and *R* is bacterial consumption

1 $$\frac{{\partial C}}{{\partial t}} = \mathop {\sum }\nolimits J - R.$$

The equation is numerically approximated using an implicit scheme. Neumann and Dirichlet boundary conditions are represented using mirror nodes. Total bacterial consumption at one node is calculated as the sum of consumption from cells residing at the respective node.

Bacterial growth is simulated using Monod kinetics and relating biomass production to substrate consumption by stoichiometry. Although Monod’s equation describes the growth of a bacterial population, it has been used frequently to relate growth of individual cells to local nutrient concentrations within individual-based models^44,49,71–73^. Change in bacterial mass for each cell is calculated by Eq. 2, where *m*_b_ is the cell mass of bacterial cell b, *μ*_*j*_ the growth rate calculated at node *j* for the respective species, and *μ*_maint_ the cell maintenance taken as 10% of the maximum growth rate^48^. Carbon is consumed by both species according to the specific yield. Additionally, aerobes consume oxygen and anaerobes consume nitrate at ratios given by the respective stoichiometry (see Table 1 for parameter values). For aerobic cells, carbon limitation and oxygen limitation terms are included in the growth rate calculation, while carbon limitation, oxygen inhibition, and nitrate limitation terms are included for the anaerobic species as shown by Eqs. 3 and 4, respectively. Here, *μ*_max_ is the maximum specific growth rate, *C*_*n*_ the concentration of substrate *n*, *K*_*n*,lim_ the limitation coefficient of substrate *n*, and *K*_*n*,inh_ the inhibition coefficient of substrate *n*

2 $$\frac{{\partial m_b}}{{\partial t}} = \left( {\mu _j - \mu _{maint}} \right) \ast m_b,$$

3 $$\mu _{{\mathrm{aerobe}}} = \mu _{{\mathrm{max}},{\mathrm{aerobe}}} \ast {\mathrm{min}}\left( {\frac{{C_{\mathrm{C}}}}{{K_{{\mathrm{C}},{\mathrm{lim}}} + {C_{\mathrm{C}}}}},\frac{{C_{{\mathrm{O}}_2}}}{{K_{{\mathrm{O}}_2,{\mathrm{lim}}} + C_{{\mathrm{O}}_2}}}} \right),$$

4 $$\mu _{{\mathrm{anaerobe}}} = \mu _{{\mathrm{max}},{\mathrm{anaerobe}}} \ast {\mathrm{min}}\left( {\frac{{C_{\mathrm{C}}}}{{K_{{\mathrm{C}},{\mathrm{lim}}} + C_{\mathrm{C}}}},\frac{{K_{{\mathrm{O}}_2,{\mathrm{inh}}}}}{{K_{{\mathrm{O}}_2,{\mathrm{inh}}} + C_{{\mathrm{O}}_2}}},\frac{{C_{{\mathrm{NO}}_3}}}{{K_{{\mathrm{NO}}_3,{\mathrm{lim}}} + C_{{\mathrm{NO}}_3}}}} \right).$$

**Table 1:** Parameters used in the mathematical model and their source reference

Parameter	Description	Value	Unit	Reference
*D*_Citrate_	Diffusion coefficient of citrate	5.9 × 10^−^^10^	m^2^ s^−1^	^74^
$D_{\rm O_2}$	Diffusion coefficient of oxygen	2 × 10^−9^	m^2^ s^−1^	^75^
$D_{\rm NO_3}$	Diffusion coefficient of nitrate	1.7 × 10^−9^	m^2^ s^−1^	^48^
*m*_0_	Average bacterial mass	1 × 10^−15^	kg	^76^
*m*_max_	Mass at division	2 × *m*_0_/1.433	kg	^44^
*m*_crit_	Mass at cell death	0.2 × *m*_max_	kg	^44^
Y	Cell yield	0.06	kg mol^−1^ Citrate	^77^
*μ*_max,aerobic_	Maximum aerobic growth rate	6.9 × 10^−6^	s^−1^	^49^
*μ*_max,anaerobic_	Maximum anaerobic growth rate	5.4 × 10^−6^	s^−1^	^78^
*K*_Cit,lim_	Half saturation coefficient for citrate	0.05	mM	^77^
$K_{{\mathrm O}_{2},{\rm lim}}$	Half saturation coefficient for oxygen	0.0063	mM	^79^
$K_{{\mathrm O}_{2},{\rm inh}}$	Half saturation coefficient for oxygen (inhibiting)	0.0063	mM	^79^
$K_{{\mathrm {NO}}_{3},{\rm lim}}$	Half saturation coefficient for nitrate	0.0351	mM	^79^
$S_{{\mathrm O}_{2},{\rm cit}}$	Stoichiometry between oxygen and citrate	2.2	mol O_2_ mol^−1^ Citrate	^80^
$S_{{\mathrm {NO}}_{3},{\rm cit}}$	Stoichiometry between nitrate and citrate	10	mol NO_3_ mol^−1^ Citrate	^77^
*p*_0_	Unbiased tumbling probability	0.25	s^−1^	^47^
χ	Chemotactic sensitivity	2 × 10^−8^	m^2^ s^−1^	^30^
ν	Corrected cell velocity for one-dimensional movement	2	m s^−1^	^47^

Cells can divide if reaching a mass at division where all properties are inherited and cell mass is divided approximately equally. Cells die if their mass reaches a critical value and are removed from the simulation.

Bacterial motility is simulated as a biased run-and-tumble mechanism^47^ including chemotaxis towards multiple nutrient and oxygen sources^30^. In each iteration, the probability of tumbling for individual cells is calculated by Eq. 5, where *p* is the tumbling probability, *p*_0_ is the tumbling probability in the absence of chemical attractors or repellents, *χ* is chemotactic sensitivity, *v* the cell swimming velocity, *μ*_max_ the maximum growth rate of the species, and $\Delta \mu /\Delta x$ the growth rate gradient with respect to the change in cell position. The calculated tumbling probability is then used to determine if the cell tumbles or not. If tumbling within a channel, there is a 50% probability to revert direction. Having reached a junction, bacterial cells are forced to tumble where a new direction is chosen at random from all connected channels

5 $$p = p_0{\mathrm{e}}^{ - \frac{\chi }{{2v\mu _{{\mathrm{max}}}}}\frac{{\Delta \mu }}{{\Delta x}}}.$$

Subsequently, bacterial cells move in a straight line at a constant velocity along a channel simulating a run. Therefore, the change in bacterial cell position is calculated by Eq. 6, where ${\boldsymbol{X}}$ is the position vector in two dimensional Euclidean space at time point *t* or *t*+1, ${\mathbf{v}}$ the cell velocity vector in direction of the channel, and Δ*t* the time step

6 $${\mathbf{X}}_{t + 1} = {\mathbf{X}}_t + {\mathbf{v}} \ast \Delta t.$$

Within each time step, the model first calculates chemical diffusion of substrates and consumption from the previous time step, bacterial motility, growth of bacterial cells, and new consumption, and finally checks for cell division and cell death.

### Model parameters and boundary conditions

Congruent to the experimental protocol, bacterial cells were inoculated at the central port combined with a constant carbon and nitrate source. A constant concentration boundary of 10 mM citrate and nitrate was applied. Oxygen was supplied from the peripheral ports by keeping a constant concentration determined by Henry’s law (0.27 mM). Bacterial consumption represents the only sink for nutrients within the network (i.e., no physical sinks). Different ratios of aerobes to anaerobes and total combined cell numbers were simulated for each network topology. The different initial population ratios, where the first number represents introduced aerobic cells and the second anaerobic cells, were 1,000 × 1,000, 500 × 500, 100 × 100, 1 × 1, 500 × 100, 100 × 500, 1000 × 1, and 1 × 1,000. The total simulated time was 7 days at 10 s time steps. All parameters of the model are obtained from literature (see Table 1 below for the original references) and are thus independent from the experiments.

### Code availability

Computer code used within this study is available from the corresponding author upon reasonable request.

### Data availability

The experimental and simulation data that support the findings of this study are available from the corresponding author upon reasonable request. CAD files containing the full description of the micrometric pore networks are available upon request.

## Electronic supplementary material

Supplementary Information
