# Supplementary material for: Spatial organization of bacterial populations in response to oxygen and carbon counter-gradients in pore networks
Source: Nat Commun. 2018 Feb 22;9:769. doi: 10.1038/s41467-018-03187-y (PMC5823907; doi:10.1038/s41467-018-03187-y)
Supplement: Supplementary file 1 — Supplementary Information [file 41467_2018_3187_MOESM1_ESM.pdf]

Spatial organization of bacterial populations in response to oxygen and carbon counter-gradients in pore networks

Supplementary Material

Borer et al.

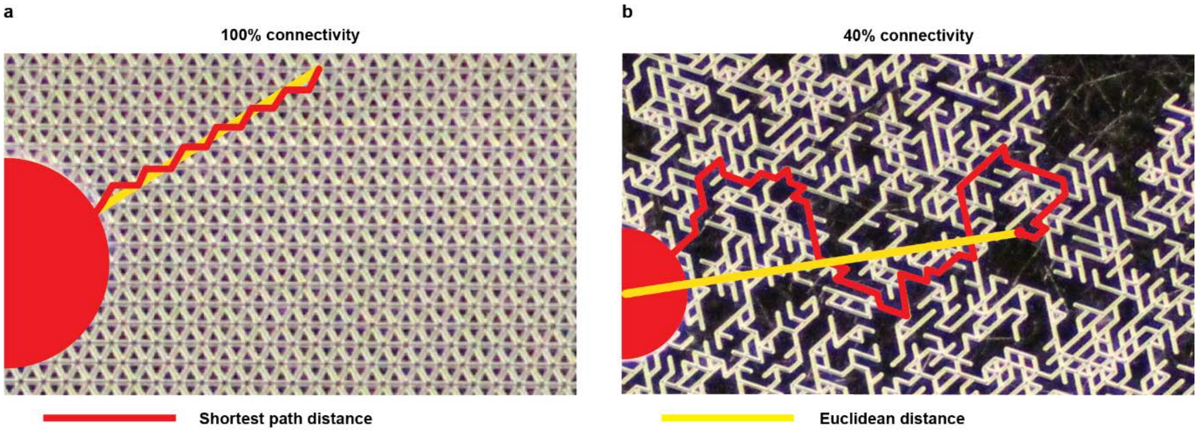

Supplementary Figure 1. Comparison of Euclidean distance vs shortest paths within the network topologies. Whilst the two metrics are similar for fully connected networks (100% connectivity, a), the difference becomes apparent in the case of the 40% lattice (b). This underlines the importance to represent location of bacterial cells with respect to the shortest path distance and not Euclidean distance.

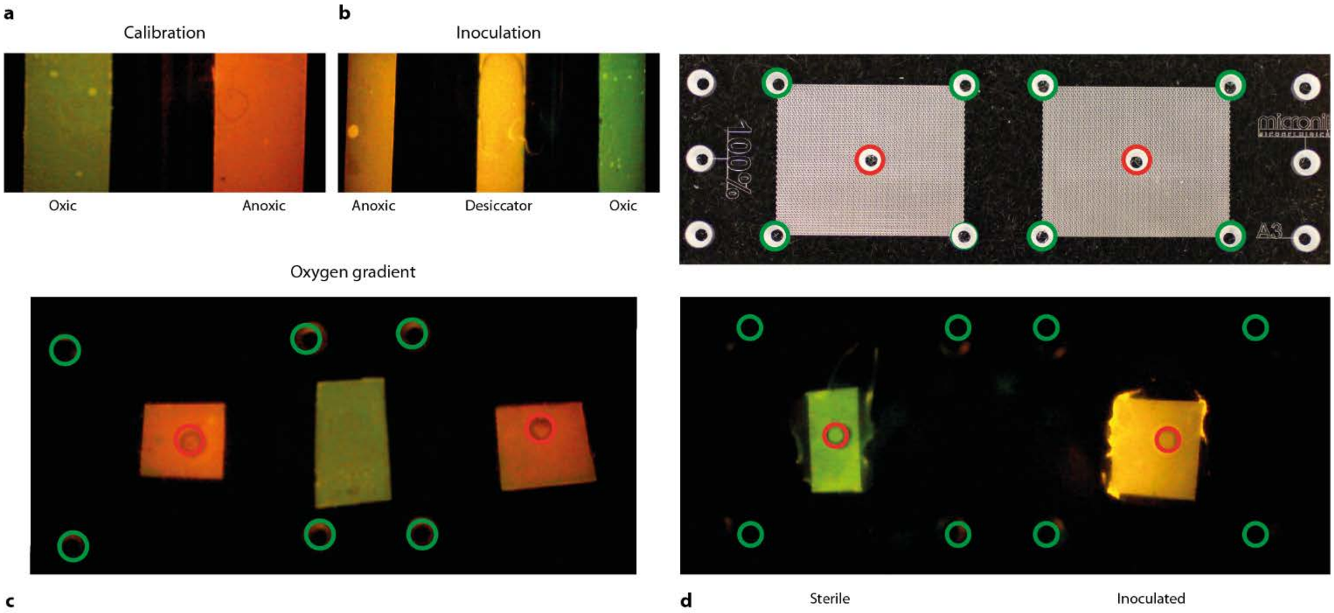

Supplementary Figure 2. Visualization of oxygen concentrations at the central and peripheral ports of the pore network. (a) A PreSens optode (VisiSens TD 1 with AnalytiCal 4 software) was used to visualize differences in oxygen concentration by means of a color gradient ranging from green (oxic) to red (anoxic). (b) Comparison of oxygen levels between calibration solutions (oxic water and anoxic sodium sulphite as recommended by PreSens) with growth media taken directly after 24h within the desiccator (used for saturation of pore networks before inoculation), demonstrating micro aerobic conditions at inoculation. (c) Difference in oxygen levels at the central versus peripheral ports after 7 days of incubation (both species) at room temperature (~23 °C) with counter-gradients, providing evidence for anoxic cores and peripheral oxygen inducing oxygen gradients within the network (experimental setup the same as replicates used for visualization of pre scale segregation in Figure 4). (d) Comparison of oxygen levels at the centre of two fully connected networks after 7 days of incubation with one lattice being sterile whilst the other was inoculated with the two species at a ratio of 1:1, providing evidence that microbial activity is required to maintain oxygen gradients within the network and resulting anaerobic niche.

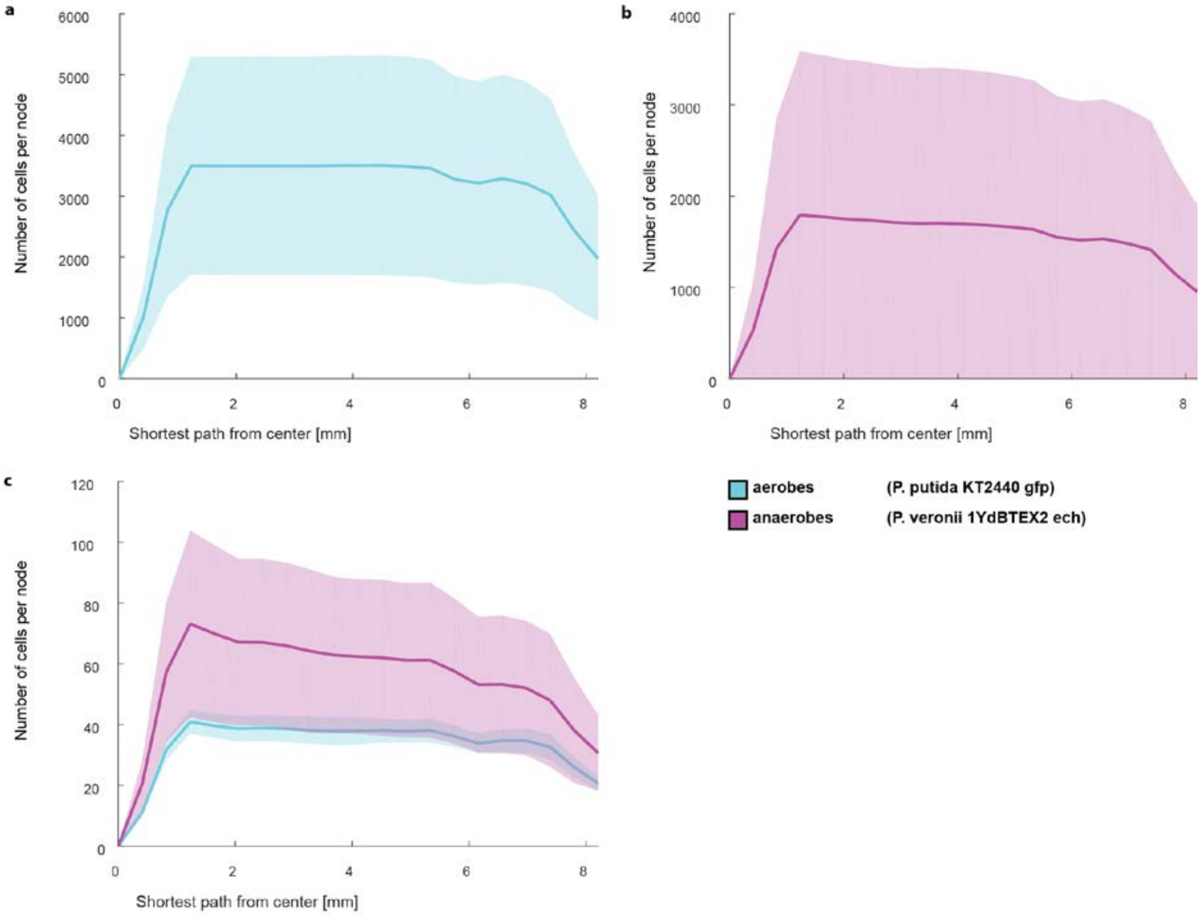

Supplementary Figure 3. Additional simulations to elucidate the importance of factors governing spatial self-organization. Simulation within fully connected pore networks in with unlimited carbon and oxygen (a) and unlimited carbon (absence of oxygen) (b) were conducted to elucidate the importance of persistent nutrient gradients on spatial self-organization. The line is the mean of all realizations whilst the shaded area represents 95% of all cells. Aerobes dominate the networks in presence of both oxygen and carbon (a) whilst anaerobes proliferate in the absence of oxygen (b). High cell density is due to the abundance of nutrients and rapid replenishment. In the absence of chemotaxis with cross gradients of oxygen and carbon (c), no segregation is observed due to the inability of bacterial cells to proliferate within their preferential habitats. Small population sizes reflect low growth rates of many cells residing in poor nutrient conditions.

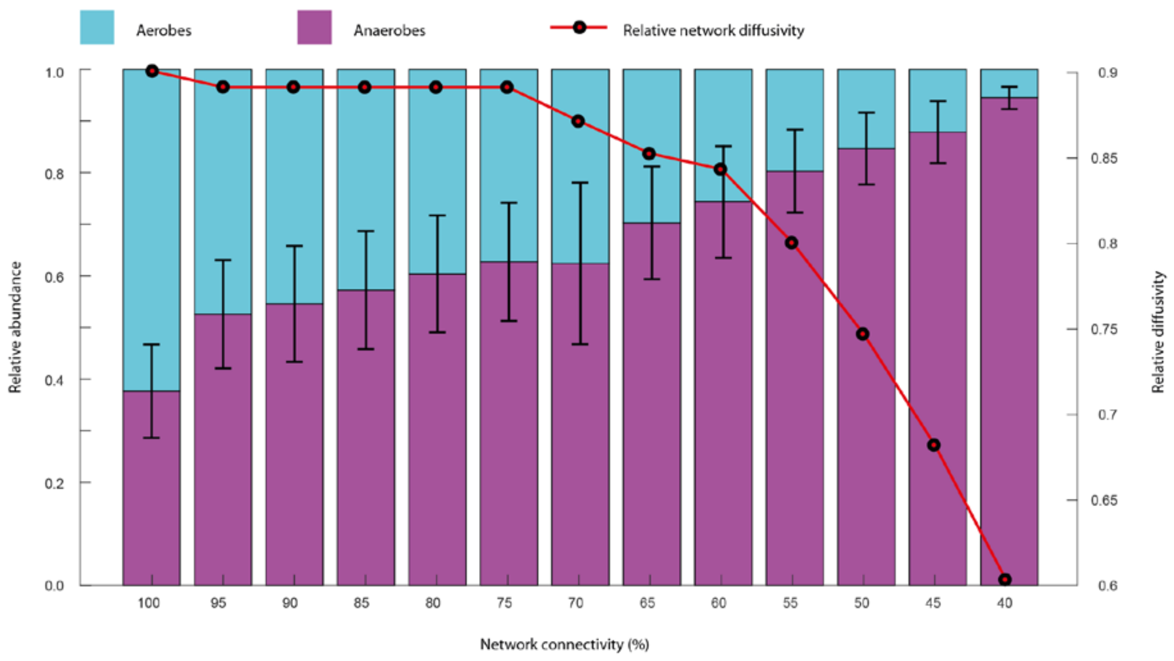

Supplementary Figure 4. Simulations to elucidate the interaction between network connectivity and relative abundance of the two species. Additional geometries with varying connectivities were created (100%, 70% and 40% geometries were taken from the experimental layout). Error bars represent one standard deviation around the mean. Boundary conditions, bacterial species and inoculation ratios are congruent to the simulations used for the main simulations. Relative diffusivity is calculated as the ratio of diffusion in pure water relative to the network diffusion including tortuous pathways for a substrate with a source at the center and sinks at the periphery. A shift in relative abundance is apparent at approximately 70% connectivity, where diffusional constraints based on network topology become significant. The 100% connected network is comparable to diffusion in pure water as it is radially homogeneous and diffusivity only marginally reduced.

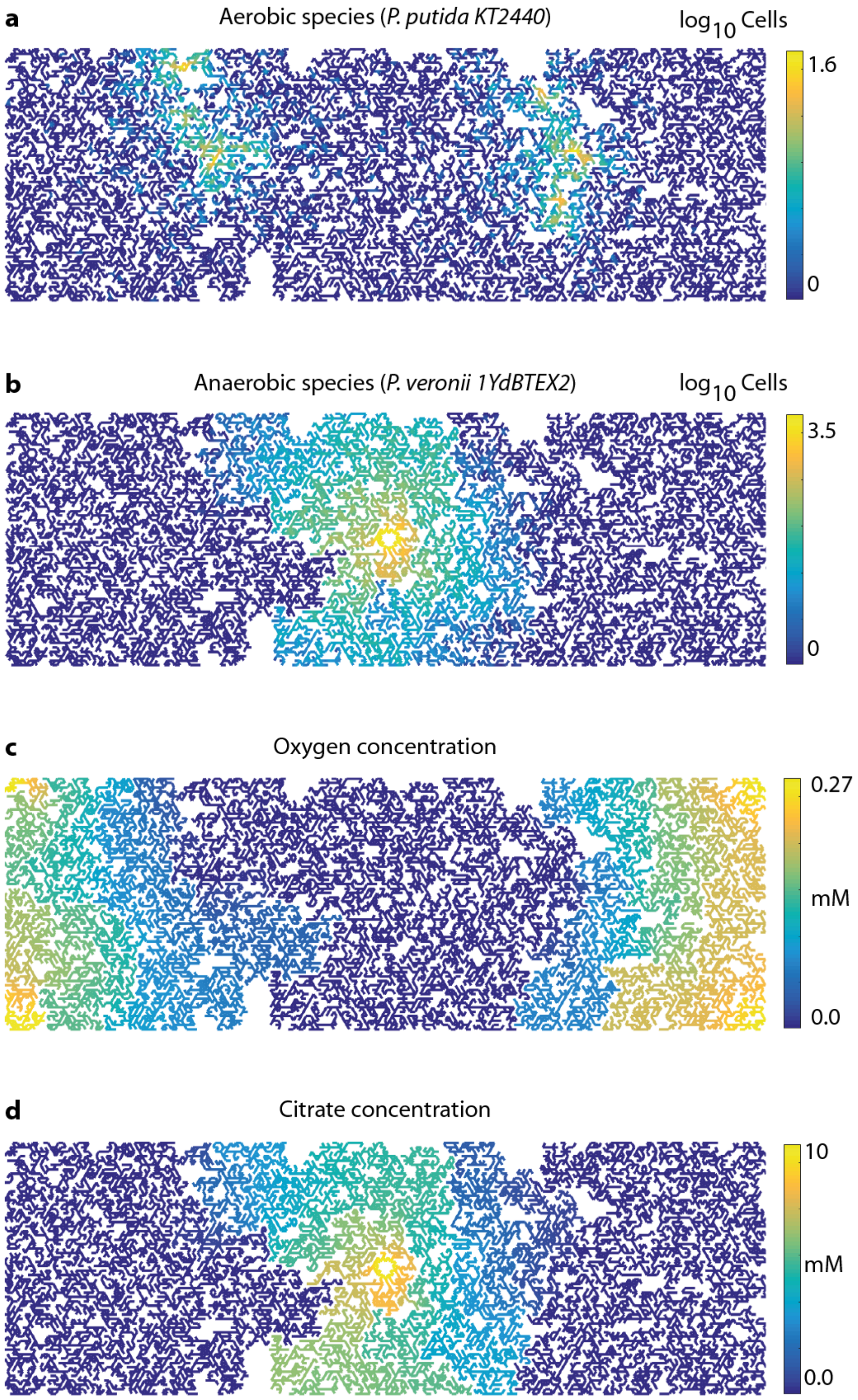

Supplementary Figure 5. Mathematical model predictions of bacterial spatial distribution and corresponding oxygen and citrate concentration fields in the pore network. Prediction of bacterial patterns for aerobes (a) and anaerobes (b) in a poorly connected (40% connectivity) lattice after 7 days of simulated time with an inoculum of 1000 cells (initial aerobe to anaerobe ratio is 1:1). Facultative

anaerobes proliferate close to the carbon-rich center (d) whilst obligate aerobes aggregate close to diffusive bottlenecks separating the oxygen-rich periphery (c) and carbon-rich center. Due to the different location of bottlenecks, spatial patterns of aerobes along the shortest path from the center are more heterogeneous in the poorly connected lattices (Fig. 4).

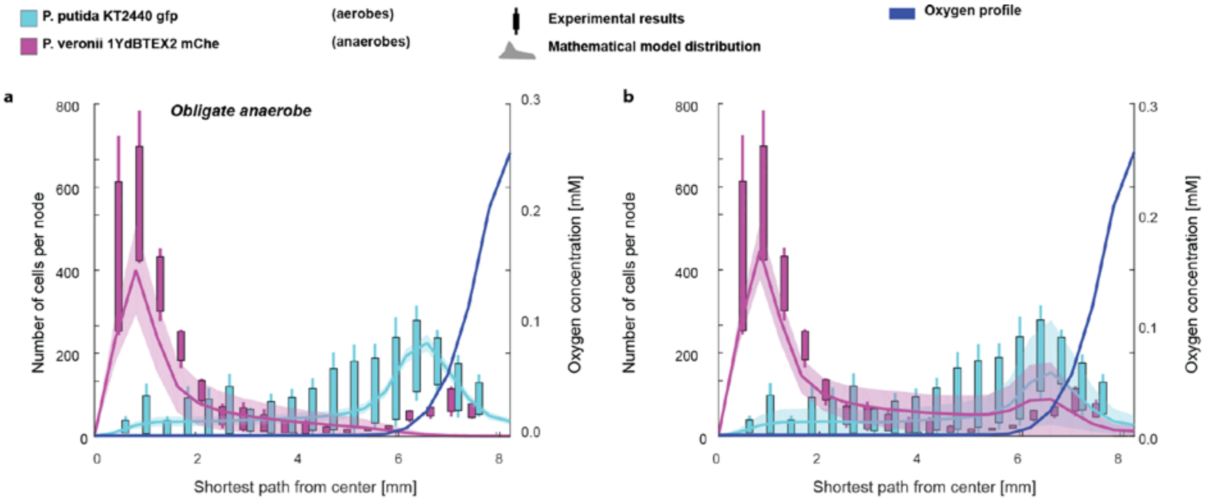

Supplementary Figure 6. Comparison of spatial segregation and oxygen concentration profile between two model versions. Prediction of spatial segregation of aerobes and (facultative) anaerobes along the shortest path from the central to the peripheral ports when *P. veronii 1YdBTEX2* is represented as a obligate anearobe (a) and facultative anaerobe (b). Experimental results are represented in boxplots (whiskers indicate minimum to maximum value of data) of three, four and five experimental replicates for the 100%, 70% and 40% pore networks, respectively. In the mathematical model results, thick line indicates the mean and shaded area includes 95% of all cells. Although a shift concerning species distribution and relative abundance is apparent, the main driver for spatial segregation, being the underlying nutrient profile (shown as oxygen concentration), remains unchanged.
